# P-902. Descriptive Study of Bridging with Caspofungin to Therapeutic Mold-Active Triazoles

**DOI:** 10.1093/ofid/ofaf695.1108

**Published:** 2026-01-11

**Authors:** Olivia Zdradzinski, Vasilios Athans, Shawn Binkley

**Affiliations:** Hospital of the University of Pennsylvania, Philadelphia, PA; Hospital of the University of Pennsylvania, Philadelphia, PA; Hospital of the University of Pennsylvania, Philadelphia, PA

## Abstract

**Background:**

Posaconazole (POS) and voriconazole (VOR) are triazoles used for mold prophylaxis and treatment. Therapeutic drug monitoring (TDM) is recommended for VOR and POS due to pharmacokinetic variability and established exposure-response and -toxicity profiles. At our institution, “antifungal bridging” is the practice of initiating caspofungin (CAS) with POS or VOR for mold prophylaxis or treatment to provide secondary coverage while pending therapeutic triazole level. The utility and stewardship implications of bridging have not been established.

**Methods:**

Retrospective single cohort of adults who received CAS and POS or VOR for ≥48 hours from 9/2021-10/2024. Patients were excluded if a triazole level was not obtained or if antifungals were intended for dual therapy rather than bridging. Endpoints were duration of therapy, time to goal level, appropriate level timing (trough/steady state), in-hospital mortality, fungal culture positivity post-discharge, and length of stay (LOS). Study variables included demographics, comorbidities, antifungal characteristics, TDM results, and microbiology data.

**Results:**

462 patients were screened with 92 encounters (25 VOR,67 POS) included. Mean age was 60 years with 49% and 40% having a history of hematologic malignancy and solid organ transplant, respectively. Most patients (91%) had infectious diseases consult. 66% of patients were receiving an antifungal prior to bridging, most commonly for prophylaxis. Treatnent was the prevalent indication for bridging, with a median bridging duration of 7 days. Median time to first level was 4 days with 80% within goal range. Many levels were not troughs or were collected prior to steady state. For initial levels not at goal, goal range was achieved within 14-21 days. On average, CAS was continued for 2 days for POS and 3 days for VOR post-therapeutic level. Median LOS was 35 days with low incidence of fungal culture positivity post-discharge. In-hospital mortality occurred in 28% patients and 38% expired within 6 weeks of bridging initiation.

**Conclusion:**

Antifungal bridging led to excess CAS durations which were partially driven by suboptimal triazole TDM. While this study was not comparative, mortality was high despite bridging therapy. Development of local guidance and further study is warranted.

**Disclosures:**

Vasilios Athans, PharmD, BCIDP, Astellas Pharma: Advisor/Consultant

